# Totipotency, development, and chromatin

**DOI:** 10.1101/gad.350470.123

**Published:** 2023-01-01

**Authors:** Maria-Elena Torres-Padilla

**Affiliations:** Institute of Epigenetics and Stem Cells (IES), Helmholtz Zentrum München, D-81377 München, Germany; Faculty of Biology, Ludwig-Maximilians Universität, D-81377 München, Germany

It has taken me a long time to figure out how to start writing this piece. I want to draw attention to the words in the title, give a personal perspective, and remain scientifically focused. This is probably too ambitious, and it is unlikely that I will achieve it.

Our research is embedded fully in the three words I chose for the title, which also reflect what drives us; that is, to reach a deeper understanding of totipotency, development, and chromatin, as well as, of course, the underlying processes and implications. The time window that encompasses the earliest developmental stages at fertilization up until the emergence of the first lineages in the mammalian embryo is fascinating for many reasons.

Primarily, it is because there is an intense period of chromatin remodeling during the first few cell divisions after fertilization that must occur hand in hand with major changes in cellular plasticity, involving regulation of the transcriptome and epigenome that also change with each individual cell division. Setting up the developmental program after fertilization also requires the remodeling of the chromatin from the gametes, which in mammals come with very different flavors: While the sperm DNA is mostly packed in protamines, the oocyte is packed into a nucleosomal chromatin configuration, albeit with very distinctive features compared with somatic cells. Over the years, the realization that the germline and preimplantation embryonic chromatin have very specific features (for example, the presence of broad domains of H3K4me3 or the absence of a mature, repressive H3K9me3-marked heterochromatin) has become consolidated. In addition, the cells in the early embryo are totipotent; that is, individual cells have the capacity to form a new being on their own, although totipotency is only transient, as cells lose this capacity during the divisions taking place before implantation, which culminate with the emergence of pluripotency.

How chromatin regulation emerges in development and whether it impacts the most notable features of totipotency and reprogramming are thus very exciting and rich topics of research. However, addressing these topics, as with many others in biology, is not straightforward. As someone who has worked at the crossroads of fields in biology, it has often been difficult to open up a path between two fields. For the “true” developmental biologists, our work was not considered as “proper developmental biology,” primarily because we rarely use genetic approaches. For the chromatin field, our work was not quite there because we cannot do “true” biochemistry. While the latter has progressed, particularly with the development of low-input approaches to study chromatin from a genomics standpoint, limitations to access mechanism still persist through many developmental model systems. *Genes & Development* has offered an excellent home for such work and, in general, for the community's efforts to add molecular understanding to developmental processes. Even when the answers to these problems may not be fully developed, the open-mindedness of *G&D* has enabled an expansion of interest to unexpected questions or to different ways to answer them, which I believe has been vital.

Looking back over the last 10–15 years, I believe that major advances to move the field forward have been made by incorporating molecular and mechanistic understanding into genetics approaches to developmental biology. Experimental embryology has also provided additional viewpoints of our understanding of early development. For example, acute manipulations of chromatin and chromatin modifiers at specific developmental stages have taught us about the requirement of specific pathways for particular developmental processes. Global demethylation of H3K4me3 by expressing the Kdm5b demethylase specifically in the mature oocyte actually results in increased expression, a quite unexpected finding that would have been difficult to pinpoint otherwise. Imaging approaches to mouse embryos have also provided new information on chromatin dynamics, for example, and have revealed that chromatin components at the earliest stages of development are highly dynamic. We also learned that repositioning of heterochromatin affects its transcriptional output, which indicates that embryonic nuclei are functionally regionalized from very early on. The initial reports that early embryos express retrotransposons and that such retrotransposons can form chimeric transcripts with the host—several of which were in fact published in *G&D*—have also given rise to a major new field in which the conception of transposable elements and their remnants has changed from being considered as passive transcriptional by-products to major players in development. This refers to both in terms of molecular regulation and also considering their impact for species propagation and evolution.

Where next? Understanding the biology of developmental systems, from the molecular standpoint, has much to offer for the years to come. The fascination of the earliest developmental stages—by their plasticity and by their unique makeup—will continue to drive mine, and many others’, curiosity and commitment for research. The current synthetic developmental biology advances—whereby “synthetic embryos” can be generated to recapitulate many features of the corresponding developmental process—will provide further biochemically tractable systems to better understand and manipulate the molecular foundations of cell decision-making, morphogenesis, and body axis formation. Complemented by parallel systems to approach stem cell and totipotent cell biology, we will be able to deepen our molecular understanding of epigenetic reprogramming, with the aim of embracing the specific nature of the underlying regulatory mechanisms to advance from a broad understanding of the epigenome into more subtle, specific regulation. This will help us to better design epigenetic targeting and manipulation approaches for reprogramming, for example, and perhaps for directing developmental pathways “à la carte.”

Publishing-wise, at times when revisions seem far too excessive and Editorial handling times appear slow and cumbersome, *G&D* has been a role model, in my experience: fast and clear, for both “yes” and “no.” We need to continue to strive for a better processes. Thank you, Terri, for your long-standing contributions toward that.

